# Deep repeat resolution—the assembly of the Drosophila Histone Complex

**DOI:** 10.1093/nar/gky1194

**Published:** 2018-11-26

**Authors:** Philipp Bongartz, Siegfried Schloissnig

**Affiliations:** Heidelberg Institut für Theoretische Studien, Schloß-Wolfsbrunnenweg 35, 69118 Heidelberg, Germany

## Abstract

Though the advent of long-read sequencing technologies has led to a leap in contiguity of *de novo* genome assemblies, current reference genomes of higher organisms still do not provide unbroken sequences of complete chromosomes. Despite reads in excess of 30 000 base pairs, there are still repetitive structures that cannot be resolved by current state-of-the-art assemblers. The most challenging of these structures are tandemly arrayed repeats, which occur in the genomes of all eukaryotes. Untangling tandem repeat clusters is exceptionally difficult, since the rare differences between repeat copies are obscured by the high error rate of long reads. Solving this problem would constitute a major step towards computing fully assembled genomes. Here, we demonstrate by example of the Drosophila Histone Complex that via machine learning algorithms, it is possible to exploit the underlying distinguishing patterns of single nucleotide variants of repeats from very noisy data to resolve a large and highly conserved repeat cluster. The ideas explored in this paper are a first step towards the automated assembly of complex repeat structures and promise to be applicable to a wide range of eukaryotic genomes.

## INTRODUCTION

*Drosophila melanogaster* has been one of the most important model organisms for over a hundred years (1). It has also played a crucial role as a proof of concept (2) for the technologies and algorithms that lead to the successful Human Genome Project (3,4). Despite the successful assembly of the Drosophila genome, the Drosophila Histone Complex has eluded assembly by conventional assembly methods (5). It consists of over a hundred copies of a 5 kbp repeat (6) that contains the coding information of the four core histones (H2A/B, H3 and H4) as well as the linker histone H1. The fast expression of histone proteins is essential for DNA replication and cell division. Therefore, eukaryotes always contain multiple clustered copies of the histone coding sequence.

The Drosophila Histone Complex contains tandemly repeated (7), highly conserved coding sequences in an unusually high number of copies. It thus represents a particularly challenging instance of a structure that occurs in similar configurations in most eukaryotes, including humans.

Therefore, it provides a reasonable basis for investigating the resolution of repeat clusters.

The development of third generation sequencing technologies like the Pacific Bioscience single molecule real-time sequencing (8–10), and more recently, Oxford Nanopore Technologies nanopore sequencing (11), has revolutionized *de novo* genome assembly. With read lengths exceeding 10 kbp, the assembly of bacterial genomes can now be regarded as a solved problem (12). In addition, for eukaryotic genomes the contiguity of *de novo* assemblies has risen by orders of magnitude, due to the possibility to span interspersed repeats with the support of unique flanking sequences.

However, while genome assemblers like FALCON (13), MARVEL (14) and Canu (15) have solved the problem of arranging millions of long reads into long contiguous assemblies, tandemly arrayed repeats still generally remain unresolved. In particular, high quality *D. melanogaster* long read assemblies have been created with each of these assemblers, but the Drosophila Histone Complex is not resolved in any of them.

The resolution of tandemly arrayed repeats is hindered by the occurrence of further repeat copies as flanking sequences. This compels us to distinguish the slightly distinct copies of the tandem repeat and to order copy versions between the unique flanking sequences of the complex. The various copy versions of the repeated sequence have to be classified by identifying the large-scale variations (i.e. indels >100 bp) and single nucleotide variants (SNVs) that characterize each copy.

Here, we introduce a novel correction heuristic based on artificial neural networks. Our heuristic decreases the error rate in extracted SNVs to a point, where automatic assembly of the complex becomes feasible.

## MATERIALS AND METHODS

### Data

To test our correction heuristic, we use several repeat datasets. A dataset of reads sampled from the histone complex, a simulated repeat dataset and 17 transposon datasets (see Table 7 for an overview). Each dataset is constructed around a repeat sequence template. The templates are consensus sequences of the repeat characteristic of the dataset.

For the histone dataset the template is the histone coding sequence (6). It is used to extract ∼5000 reads that contain instances of this sequence via mapping from a high quality PacBio dataset sequenced from a subline of the ISO1 (y;cn,bw,sp) strain of *D. melanogaster* (16). We extract all reads that align to the template over 1 kbp with an alignment error below 30%. This dataset has a coverage of >90× and an average read length of >10 kbp.

To independently benchmark our correction heuristic, we extract reads containing transposable elements from the same sequencing run to build 17 different transposon datasets. The extraction is again done via mapping, with a minimal local alignment length of 1 kbp and a maximal alignment error of 30%. The transposon datasets are based on templates taken from the transposon sequence canonical set (17) [https://github.com/cbergman/transposons/], see Table 1. They contain Drosophila transposons of comparable length (>4 kbp) as the histone repeat.

**Table 1.** Properties of the transposon datasets

No.	Av. length	Copies	Coverage	Gr.truth coverage
**0**	7215 bp	37	37	23
**1**	7380 bp	49	45	27
**2**	4672 bp	25	45	27
**3**	6850 bp	44	51	35
**4**	6093 bp	16	37	29
**5**	7538 bp	34	46	26
**6**	4479 bp	7	51	41
**8**	5168 bp	9	54	41
**9**	6371 bp	13	49	33
**10**	5201 bp	89	48	30
**11**	4762 bp	135	48	33
**12**	4690 bp	157	46	33
**15**	4361 bp	8	48	29
**16**	7481 bp	12	35	18
**19**	6128 bp	5	51	32
**20**	5376 bp	20	41	30
**21**	5048 bp	22	43	32

The numbering is according to the transposon sequence canonical set, missing numbers are due to transposons being below the length cut-off. Ground truth coverage is calculated on the basis of the reads that can be assigned to a cluster of flanking sequences.

Additionally, we simulate a 5 kbp repeat family with 100 copies using a simulation tool we implemented. As template for the simulation, we use an empirical sequence randomly sampled from the *Escherichia coli* reference. We then first create 100 identical copies of the template. These are then perturbed via a pool of 300 random SNV, each of which we assign to a random subset of the copies. Finally, the actual simulated reads are obtained from these perturbed copies superimposing the typical PacBio error rate (11.5% insertions, 3.4% deletions and 1.4% mismatches). We use this simple version of a simulated repeat dataset to verify our correction heuristic and the associated preprocessing steps. The test on simulated data further shows that the problems solved in the processing steps are not specific to the selected empirical test datasets of the chosen genome.

In the following, we provide an overview over the individual (pre-)processing steps.

### Multiple sequence alignment

In the first preprocessing step, our objective is to subdivide each extracted histone read into instances of the repeat sequence. Some of these instances contain insertions, deletions or duplications. If properly identified, insertions, deletions and duplications, allow for assigning these instances either to unique repeat copies or to small groups of repeat copies. All other instances of the repeat sequence that do not deviate in such a significant way from the histone template, will have to be disambiguated by more sophisticated means.

**Figure 1.**
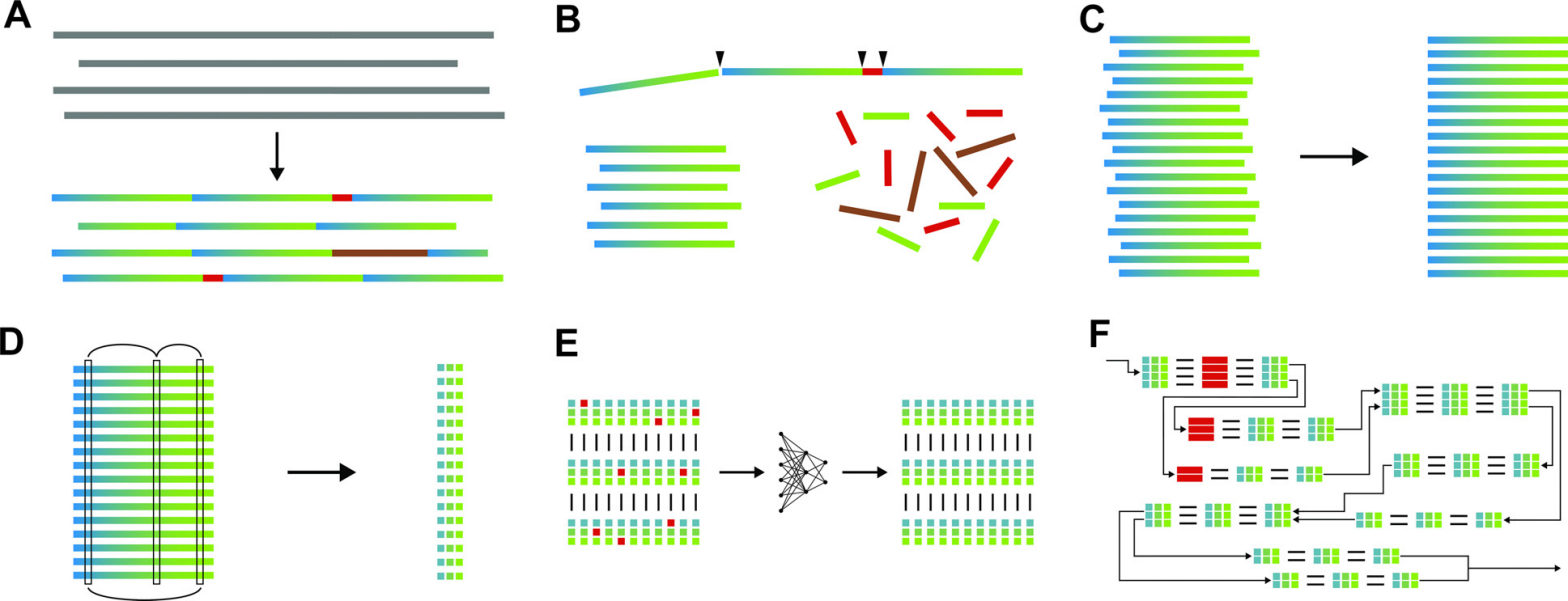
In (**A**), we illustrate the fundamental problem: a hypothetical master consensus of all copy versions is more similar to the signatures than signatures that belong to the same copy are to each other. With that property, it acts like a vanishing point, signatures with low error rate all seem to be quite similar. The neural network depicted in (**B**) solves this problem because it is able to pick up on the sub-signatures shared by signatures from the same copy. In (**C**), we show the unrolled converging corrector: the signatures are repeatedly corrected by the concatenated neural networks using them and both neighbouring signatures until the bases stop changing. Panel (**D**) shows one assembly graph greedily traversed starting from one end of the complex. Node size and number give the size of each assembly group after mapping all clusters, even those that do not fit anywhere well. This over mapping allows us to double check on over represented groups and to catch the two collapsed parts of the complex marked in red. The other coloured nodes stand for large scale variations.

To that end, we map short (100–250 bp) substrings of the histone coding sequence on each read. The mapping information is then used to detect several insertions, deletions or duplications between 100 and 8 kbp in length (see Figure 1A)). These large-scale deviations from the template uniquely classify several distinct repeat copies. We then cut all histone reads into such instances of uniquely classified copies and into all other, non-deviating instances of the histone template (6,18), see Figure 1B).

Subsequently, the non-deviating instances of the histone template are arranged into a global multiple sequence alignment (MSA). The uniquely classified instances will be used later on to provide additional information for the correction algorithm and the assembly.

The transposon reads are cut into unique flanking sequences and transposon sequences analogously. The transposon sequences are also arranged into an MSA. The flanking sequences are clustered according to alignment scores (Levenshtein distance) to provide the ground truth for copy versions. For the simulated data only repetitive sequences are generated. They can hence be immediately arranged into an MSA without any preprocessing.

All initial MSAs are built incrementally by aligning the repetitive sequences to the respective repeat sequence template. These MSAs are then refined by realigning the sequences iteratively, minimizing the unweighted sum-of-pairs score (19), see Figure 1C). Both of these tools are implemented from scratch in the C programming language.

### Identifying discriminative columns

The refined MSAs contain sequences that are sampled from highly similar but not identical repeat copies. These sequences have now been arranged in such a way that a difference between repeat copies becomes detectable as groups of different bases or alignment gaps within a column. However, not every sequence covers the entire breadth of the MSA. Additionally, the substantial error-rate (12–15%) in the original reads, introduces instances of all bases, as well as alignment gaps, into all columns, whether they contain a repeat copy difference or not.

Therefore, each column typically contains instances of all four bases $\{ {A,C,G,T} \}$, as well as alignment gaps and coverage gaps $\{ { -,\_} \}$. We define for a given MSA $M \in {\{ {A,C,G,T, -,\_} \}^{r,c}}$, with $r$ rows and $c$ columns, a given ‘base’ (including the alignment gaps) $B\ \in \ \{ {A,C,G,T, - } \}$, and a given column $i < c$, a base group $G_B^i: = \{ j < r|{M_{ij}} = B\}$. To identify columns where entries differ from the majority entry due to the discriminative variation between repeat copies (as opposed to random error), we calculate the statistical significance of the intersections between base groups from different columns $i,l$, $G_{{B_1}}^i \cap \ G_{{B_2}}^l = \ \{ {j \in G_{{B_1}}^i\ |\ j \in G_{{B_2}}^l\ } \}$.

The intersection of two base groups, induced by random error is an instance of the classical urn problem ‘drawing without replacement’. Two additional details have to be taken into account. The base groups in adjacent columns are not statistically independent, therefore, we only compare base groups from columns that are at least 40 columns apart. And given that not every sequence in the MSA will cover both columns, we have to base our calculation only on those elements of the base groups that share coverage for both columns.

To this end we use the cumulative hypergeometric probability $CHG( {G_{{B_1}}^i,G_{{B_2}}^j} ):{\rm{\ }} = {\rm{\ min}}(P( {X \mathbin{\lower.3ex\hbox{$\buildrel>\over {\smash{\scriptstyle =}\vphantom{_x}}$}} k} ),P(X < k))$, where 
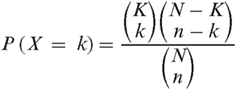
, for $N$ being the shared coverage of both groups, $K$ the size of base group 1 restricted to the shared coverage, $n$ the size of base group 2 restricted to the shared coverage and $k$ the size of the intersection between the base groups.

The CHG has been approximated by the Poisson distribution (20) in the past. Instead, we discard 99.9% of all intersections by means of the fast normal distribution, while for the remaining intersections, we calculate the CHG exactly (21).

The ubiquity of erroneous insertions (11% in PacBio data), means that discriminative insertions will share a column with a large number of false positives. This makes the detection of discriminative insertions difficult, while handling the noise in the detected discriminative insertions is even more challenging. For this reason, we restrict our detection to columns that contain a majority of bases as opposed to those containing mostly gaps. We choose a relatively high significance cutoff of negative log-probability $ - log( {CHG( {G_{{B_1}}^i,G_{{B_2}}^j} )} ) >15.0$ based on an empirical assessment via trial and error. We call a column a *discriminative column*, if the statistical significance of the intersection of at least one of its base groups with another base group is above the statistical significance cutoff.

The transposon sequences are generally less highly conserved than the histone coding sequence. Thus, we restrict the number of selected discriminative columns in our test sets to 300 to conduct an as fair as possible correction comparison to the histone dataset.

The selected discriminative columns $D$= {${d_1},{d_2},{d_3}, \ldots {d_n}$} constitute the $n$ distinguishing features, on the basis of which we disambiguate the instances of the repeat sequence.

For each row $i$ of the refined MSA $M$, we define a signature ${S_{j < n}}$ as a vector of entries $\{ {M_{id}}|\ d \in D\}$, see Figure 1D). Additionally, we create a unique faux-signature for each large-scale variation, which is likewise an element of ${\{ {A,C,G,T, - } \}^n}$. This allows us to model each read $R:[ {{S^1},{S^2},{S^3},\ \ldots {S^{{N_R}}}} ]$ as a list of signatures.

### Correction

The native sequencing error rate is expected to carry over into our extracted signatures. Additionally, the high insertion/deletion rates in PacBio reads result in an unavoidable bias. This bias arises as those frequently inserted bases often provide better options for optimizing the alignment score than the correct base. Consequently, rare variations are more likely to be treated as an error by the MSA algorithm. Furthermore, the differences between copies are distributed in a hierarchical fashion, due to an evolution of copying and mutation (22). This means that a large fraction of the copies is highly similar, if not identical. Complicating matters even more, not every read shows the average error rate. Instead, the error rate, especially the rate of insertions, varies strongly from read to read. For these reasons, successfully extracting distinguishing features from the reads does not automatically induce a correct disambiguation of repeat copies.

**Figure 2.**
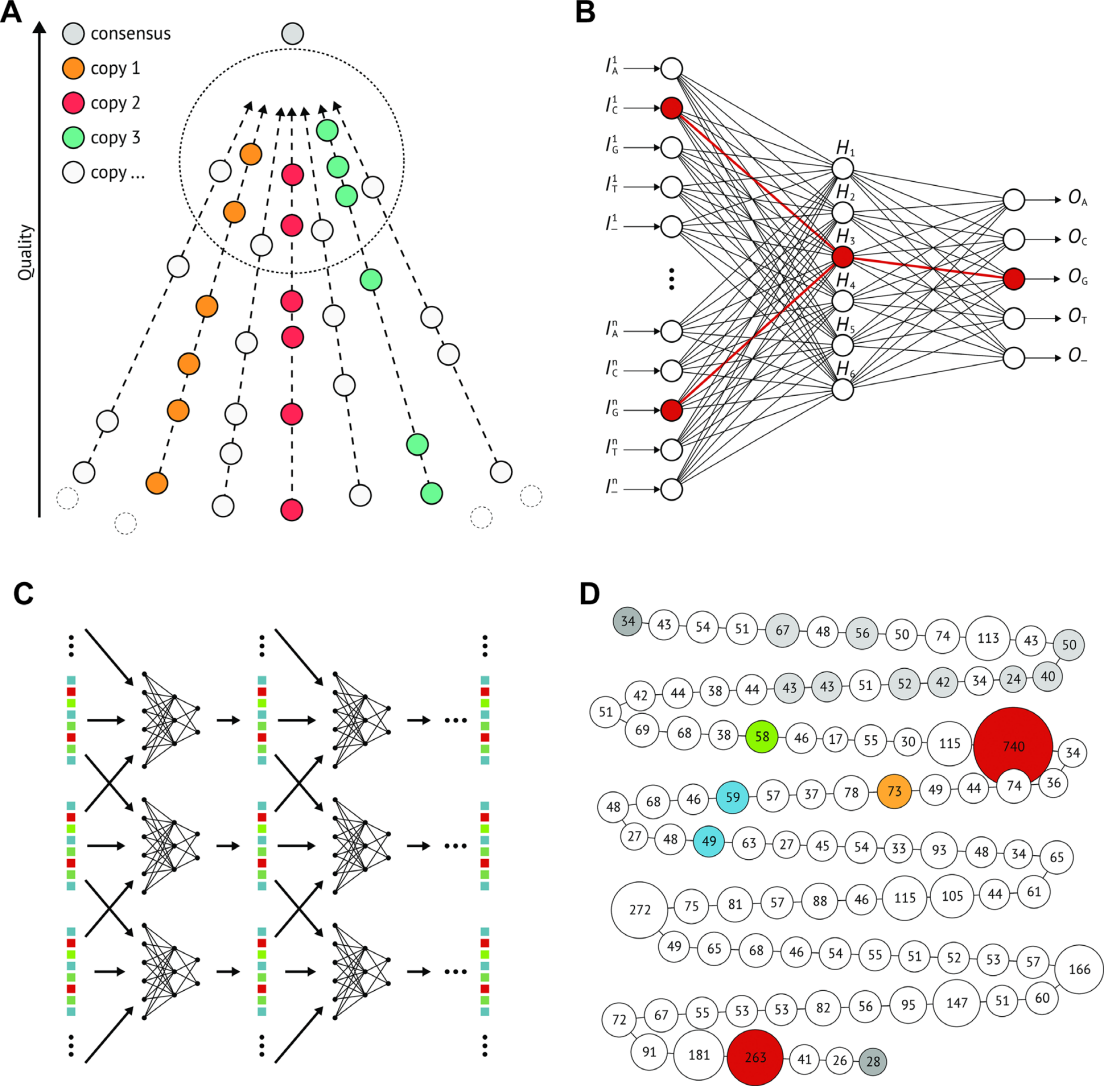
In (**A**), we examine full signatures with ground truth information. For each signature, we calculate the likelihood that the *n*-th best overlapping signature belongs to the same ground truth copy group. This likelihood starts low and drops fast, whereas the corrected signatures have a significantly higher likelihood of correct overlaps, which stays stable for the 25 best overlaps. Panel (**B**) shows the error reduction achieved by first pass correction and the converging corrector. In (**C**) and (**D**), we use a t-distributed stochastic neighbor embedding visualization (t-SNE) to show how the correction facilitates the separation of neighbouring groups of signatures.

Note that the expectation that sequences with more similar features belong to the same repeat copy does not hold. The large number of copies, the high and varying error rate, the MSA bias and the small number of differences between repeat copies lead to the situation that similarity measures between signatures are almost completely dominated by the noise level (see Figure 2A). Subreads sequenced from the same repeat copy are not characterized by having more similar features, despite on average this being the case, but rather by sharing a characteristic subset of features, which is *a priori* unknown. This misleads standard clustering algorithms or read overlapping approaches. Non-standard clustering approaches based on rare subsequences of signatures face substantial run time limitations, as the number of possible subsequences quickly grows prohibitive with increasing subsequence length.

We develop a machine learning architecture that specifically exploits the structure described above to correct signatures to a point where standard approaches become feasible. We use neural networks to utilize these underlying characteristic subsets of features. The task of these networks is to predict the instance of a target feature within a signature, based on all other entries of the signature and of the two neighbouring signatures within the same read, if available. The two neighbouring signatures provide additional information that allows the neural networks to improve prediction. However, using even more bases from the same read would impede generalization as signatures with that many neighbours become rare.

The basic structure of the neural networks we use is a simple fully connected network with one hidden layer, for a comprehensive treatment of neural networks, see (23). Every $i$-th base $B \in \{ {A,C,G,T, - } \}$ in a signature $S \in {\{ {A,C,G,T, - } \}^n}$ is encoded as a one hot vector of length five ${I^i} = ( {I_A^i,I_C^i,I_G^i,I_T^i,I_ - ^i} )$ with 
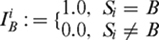
. The input vector $I$ for a given signature consists of a concatenation of the vectors ${I^{i < n}}$ of all bases of the full signature or in the histone case of the signature and its two direct neighbours, but excluding the target base ${S_j}$. The excluded target base ${S_j}$, represented by the vector ${I^j}$, is then approximated by the output vector of the neural network, $O \in ( {0,1} ){{\rm{\ }}^5}$ with $\mathop \sum \limits_{B \in \{ {A,C,G,T, - } \}} {O_B} = \ 1.0$. The output vector is calculated using a softmax function $\sigma ( {{z_j}} ):\frac{{{e^{{z_j}}}}}{{\mathop \sum \nolimits_{k\ = \ 1}^5 {e^{{z_k}}}}}$ and can be interpreted as a probability distribution over $\{ {A,C,G,T, - } \}$.

For each feature, a distinct and randomly initialized network is trained using backpropagation with gradient descent (24). For each given signature, it strives to predict the base at the chosen feature using the bases at all other features as input. Until a given accuracy is reached, the learning rate is adapted. ${L_2}$-regularization (25) is used to aid generalization. Dropout (26) can be used, but is discarded if learning stalls. If possible, we train until we reach a prediction accuracy of >97%, though some networks stall significantly below this threshold. Each trained neural network can then be used to correct its target base in every signature by changing it to the most probable predicted base.

As illustrated in Figure 2B, the hidden layer enables the network to model the sub-signatures that characterize groups of signatures that share a certain base at the target feature. The idea is that this allows the network to create generalized predictions that are closer to the underlying truth than the actual data, without being misled by overall similarity or dissimilarity of the complete signatures. Consequently, simply using linear regression would be bound to fail, while deploying more hidden layers would not improve the accuracy. This intuition behind the hidden layer can be verified by observing certain sub-signatures that fully excite neurons of the hidden layer in trained networks.

### Converging corrector

While some discriminative SNVs occur in many copies and in parallel with other SNVs, other SNVs might describe only a single specific copy and be the only SNV that does so. Therefore, the signal that has to be recognized to accurately predict the different copy versions that lead to a certain base at a certain feature varies greatly. Picking up the weakest signal necessitates an overfitting of the network to the data. This means that the network learns to predict the bases of individual signatures by recognizing their specific pattern of erroneous bases. This initially impedes generalized prediction.

By training a separate network, ${N_j}:{S_{i < n,\ i \ne j}} \to {S_j}$, for every feature $j < n$, that is part of the signatures, we create a signature to signature function by concatenation, $N:\ = { \oplus _{j < n}}{N_j}:{S_{j < n}} \to {S_{j < n}}$. A fixpoint of this function is a signature in which all bases are consistent with each other as judged by the neural networks. Intuitively, every error free signature should be internally consistent and therefore constitute a fixpoint. This suggests that one should repeatedly apply the function to a signature until convergence. In the first pass over the data, the neural network might still recognize individual signatures. Subsequent corrections can only rely on the underlying pattern, since the characteristic signature errors have already been corrected in earlier passes. This yields a generalized, instead of an overfitted correction.

Regarded on a more abstract level, the function that outputs the number of bases in a signature that are equal to the predicted base can be seen as describing a consistency landscape of signatures. Fixpoints are maxima in this consistency landscape since every base is the predicted base. The repeated application of our concatenated networks to a signature until convergence can be seen as an ascent towards the nearest consistency maximum.

### Assembly

After reducing the error rate of the signatures, we can cluster the corrected signatures and traverse the assembly graph that is given by the resulting clusters. The clustering algorithm we use on the corrected signatures is a variation of the popular k-means clustering (27).

To reach the best possible assembly result we use all available information. That includes the corrected signatures, the unique sections classified in our first preprocessing step and the results of the following two additional analyses.

There exists a shorter version (4.8 kbp) of the repeat sequence that has already been described in the literature (8). It differs from the histone template by a large deletion, that we also detect and classify in our preprocessing analysis. We create a separate MSA for this 4.8 kbp repeat. Due to the substantially lower copy number compared to the whole complex, the detected SNVs allow us to divide these shorter sequences into three different copy versions.

Additionally, we extract indels of 3 to 30 bases by clustering sections of the rows of the original MSA. These indels are challenging to detect from the extracted features alone. Both, short versions and indels are added to the corrected signatures in the form of fake triple base features. Furthermore, we extend each signature to encompass the features of all neighbouring signatures within the same read.

As centroids, we choose all extended and corrected signatures, whose coverage extends in both directions, for at least $c$ bases. The selected centroids are restricted to these $c$ bases, to ensure that all centroids have the same coverage. The parameter $c$ is empirically chosen to be 1.2 times the length of a signature. This results in an average coverage of each repeat copy by seven centroids. Thereby, it is highly unlikely that a repeat copy is missed. After this initialization, all extended signatures $S$ are distributed among the centroids$\ C$ by the first best fit according to ${\rm{D}}( {{\rm{C}},{\rm{S}}} ) = \mathop \sum \limits_{i\langle n |{S_i} \ne {C_i}} 1$. In a second round, the consensuses of these initial clusters are used as centroids. Finally, all elements of clusters under a size cut-off are distributed among the remaining clusters.

Due to the high number of initial centroids, the clustering generally splits the signatures into more clusters than there could possibly be repeat copies. These clusters ${c_j},{c_i} \in C$ naturally constitute the nodes of an assembly graph $G: = ( {C,{E_{0 < n < 3}}} )$. Two nodes ${c_i},{c_j} \in C$ are connected by a directed edge, if there exists a read $R = [ { \ldots {S^k}, \ldots,{S^{k + d}} \ldots } ]$, such that ${S^k} \in \ {c_i}$, and ${S^{k + d}} \in {c_j}$ with $d \in \{ {1,2} \}$. For a given distance $d$, the set of these directed edges is denoted by ${E_d}( {{c_i},{c_j}} )$.

Therefore, the task remaining is a layered graph drawing, in which clusters are linearly ordered into layers that can contain more than one cluster such that the order of these layers respects the order of signatures in reads as far as possible.

At each step, we choose the cluster whose best placement maximizes the scoring function $SF( {c,l} ): = S{F_1} + 2\ \times S{F_2} - S{F_3} - 2\ \times S{F_4} - S{F_5}$, where $S{F_1}: = \sum\nolimits_{{E_1}( {v,c} )|v \in {L_{l - 1}}} 1 + \sum\nolimits_{{E_1}( {v,c} )|v \in {L_{l + 1}}} 1$, $S{F_2}: = \sum\nolimits_{{E_2}( {v,c} )|v \in {L_{l - 2}}} 1 + \sum\nolimits_{{E_2}( {v,c} )|v \in {L_{l + 2}}} 1$, $S{F_3}: = \sum\nolimits_{{E_1}( {v,c} )|v \notin {L_{l - 1}}} 1 + \sum\nolimits_{{E_1}( {v,c} )|v \notin {L_{l + 1}}} 1$, $S{F_4}: = \sum\nolimits_{{E_2}( {v,c} )|v \notin {L_{l - 2}}} 1 + \sum\nolimits_{{E_2}( {v,c} )|v \notin {L_{l + 2}}} 1$, $S{F_5}: = \sum\nolimits_{v \in {L_l}} {D( {c,v} )}$, $D( {c,v} ): = \sum\nolimits_{i\langle n |{C_c}[ i ] \ne {C_s}[ i ]} 1$ D(c, v), ${C_c}$ is the consensus of cluster $c$, ${L_l}$ contains the cluster elements currently assigned to the $l$-th layer and ${E_d}( {v,c} )$ is the set of directed edges defined above. This score rewards edges consistent with the already placed clusters and punishes edges inconsistent with the earlier cluster placements, as well as differences between the cluster consensuses within a layer.

On the resulting assembly, we call a consensus sequence using the designated PacBio variant caller quiver (28). This is expected to result in a Q40 sequence for the minimal coverage of our assembly, Q45 for 97% and Q55 for 82% of the complex.

### Validation

To validate our automated assembly and to assess the accuracy of the correction algorithm, we also created a hand-curated assembly of the uncorrected signatures. In the following, we describe several insights that make manual assembly possible and the analyses based on these insights.

The expansion of the complex by unequal recombination (22), tends to create adjacent identical copies. This entails that the instances of a given distinguishing feature are likely to be clustered within the complex. Such neighbour similarity makes overlapping approaches infeasible, mainly due to the difficult statistical assessment of the trade-off between long overlaps and ‘good’ overlaps. But with a simple statistical analysis we can utilize the neighbour similarity to considerably reduce the problem size.

For each instance $b\ \in \ \{ {A,C,G,T, - } \}$ of a feature $v < n$, we define a clustering coefficient $Cc( {v,b} ): = \sum\nolimits_{S|\ {S_v} = \ b} 1 /\sum\nolimits_{S|{S_v} \ne b} 1$ for the $S \in R$ with $\sum\nolimits_{{S^{i\langle {{N_R}} |S_v^i = b}}} {1 >1}$, where each read $R$ is represented as a list of signatures $R: = [ {{S^1},{S^2},{S^3},\ \ldots {S^{{N_R}}}} ]$, which is simply the number of occurrences of an instance divided by the number of non-occurrences of the instance over all reads in which it occurs at least twice.

Intuitively, this number captures in how many contiguous parts the subcomplex described by the instance $b$ occurs within the complex, that is, how strongly the copies belonging to the subcomplex are clustered. The problem can now be broken down into smaller locally connected sub-clusters. This is done by selecting subsets of reads that are defined by the occurrence of an instance. We then manually analyse instances in order of decreasing clustering coefficients.

In each of these smaller problems we try to identify combinations of features that appear to describe a single repeat copy. Each of these combinations of features defines a fixed group of signatures in which it occurs. These fixed groups are connected if their signatures occur in the same reads. They are strongly and consistently connected if their signatures occur often and with a consistent distance in the same reads. To each side, a third of signatures has a full signature neighbour. Assuming a coverage of $C$ and an independent assignment of signatures to two fixed groups of size ${C_1}$ and ${C_2}$, we can expect $\frac{{{C_1} \times {C_2}}}{{C*{3^d}}}$ consistent connections between them, if they accurately describe copies that have a distance $d$ in the complex. We use this observation to validate independently described fixed groups. We cannot always define unambiguous groups that are just one or two copies away from each other. These gaps have to be filled with long reads that can be anchored in validated fixed groups.

By this slow manual process, the whole complex can be assembled. This manual assembly constitutes the ground truth for the automated assembly described above.

To assess the results achieved by our correction heuristic, we use transposon reads from the same dataset, for which the ground truth is provided by unique flanking sequences, as well as a simulated dataset. For the histone dataset the ground truth is given by the manual assembly described above.

To quantify the results of the presented correction heuristic, we use the following metrics that are calculated for those signatures that are assigned to a ground truth group. For both, the first pass of the correction, and the fully converged correction, each signature has a corresponding corrected signature, a ground truth consensus of uncorrected signatures (consensus) and a ground truth consensus of corrected signatures (cor-consensus).

The ‘error’ is the percentage of bases of the signatures that differ from the respective consensus. We report the percentage of bases of the cor-consensuses that differ from the consensuses as ‘collapsed variations’. ‘Internal consistency’ denotes the similarity of uncorrected signatures to the consensus and corrected signatures to the cor-consensus, respectively.

‘Recall’ is the percentage of true positives among the correct positives according to the consensus, or 100% - ‘error’ as defined above, whereas ‘precision’ is the percentage of true positives among the positives.

## RESULTS

### Assembly

The greedy layered graph drawing algorithm can in principle be started with any cluster. Here, the flanking sequences and the unique large-scale variations are the obvious initial choices. Depending on the starting cluster our automated assembly algorithm correctly orders up to 90 consecutive copies out of 113 (including flanking sequences and large-scale variations).

Starting from the left end of the complex and for most other unique large-scale variations as starting cluster, our automated assembly algorithm only fails at two histone complex locations. Around repeat copy 32, a combination of two copy versions occurs twice, one after another, and is not resolved by the clustering. Also at the right end of the complex the copies are extremely similar and therefore remain unresolved. Between these locations and the flanking sequences, 35, 60 and 3 of the copies are correctly arranged. Figure 2D shows the assembly graph resulting from a graph traversal starting from the left end of the complex. The red nodes indicate misassemblies consisting of collapsed copies.

The combination of clustering and greedy layered graph traversal places 90% of the signatures into a layer which contains a majority of signatures from the same copy according to the manual assembly, resulting in almost identical consensus signatures.

### Correction

**Figure 3.**
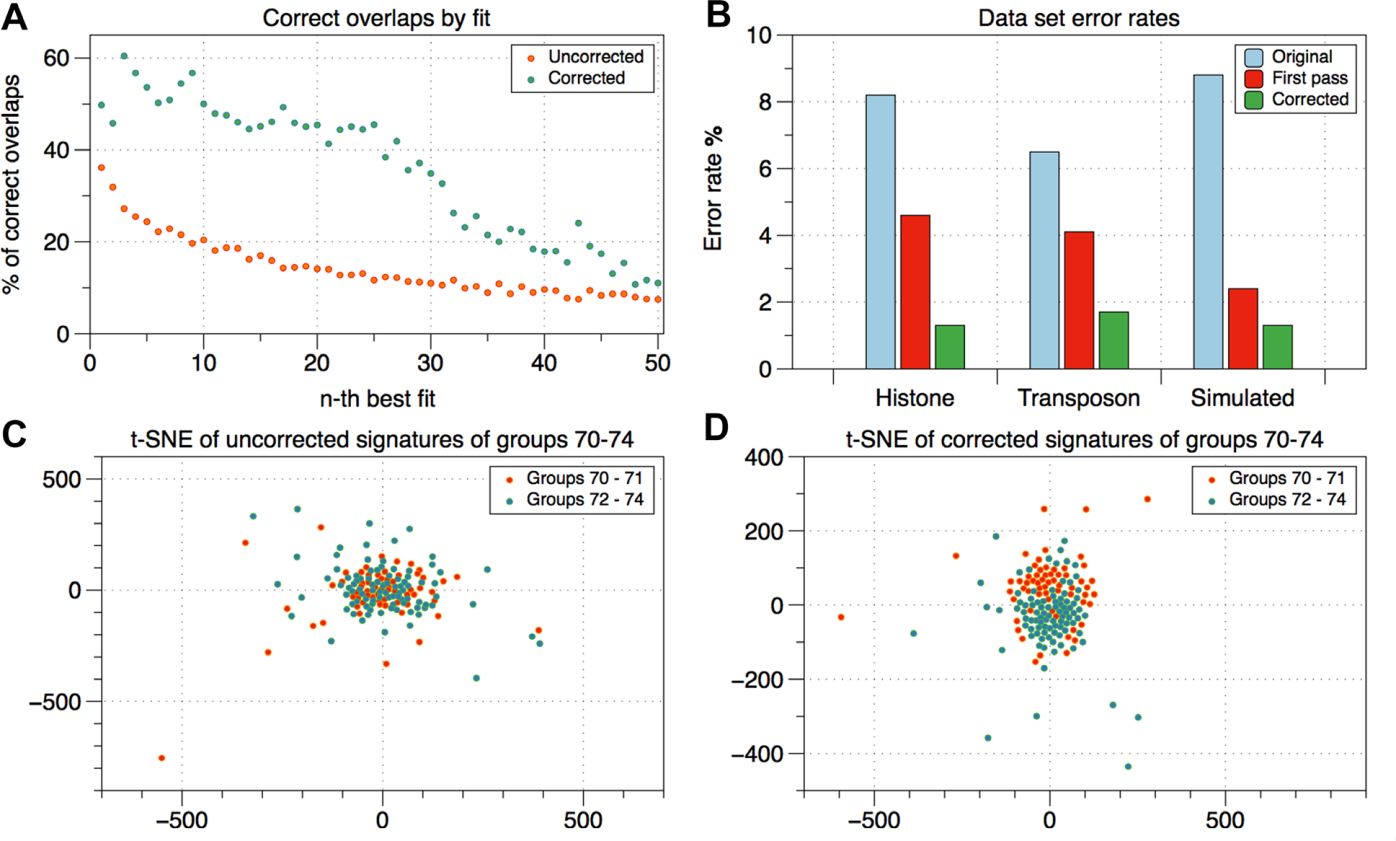
Panel (**A**) shows how raw sequencing data is categorized as repeat or unique sequence using the mapping information of subsequences of the repeat template. In (**B**), the reads are cut and the repeat sequences are arranged into a MSA. Panel (**C**) shows the refinement of the MSA. In (**D**), corrections between rows are detected and statistically significant bases are collected into signatures. Panel (**E**) illustrates how the signatures are corrected via neural networks. In (**F**) finally, the signatures are clustered and the resulting assembly graph is traversed.

Table 2 shows that we achieve significant error reduction in all datasets. The comparison with Table 3 shows that the converging corrector leads to a substantial accuracy improvement compared to the accuracy achieved in the first pass. In our target dataset, the Drosophila histone sequences the error of 8.22% is reduced in the first pass to 4.64%. This is then further reduced to 1.32% in the converging corrector. Figure 3 illustrates how this error reduction translates into substantially improved overlap accuracy and ‘sharper’ differences among signature groups. It is worth noting that reinserting the corrected SNVs into the reads would not substantially change the read error. Our correction heuristic solely intends to ‘sharpen’ the extracted differences between repeat copies.

**Table 2.** The error rate reduction of the converging corrector

Datasets	Original error	Corrected error	Collapsed variations
**Histone**	8.22%	1.32%	0.42%
**Transposons(median)**	6.51%	1.78%	0.41%
**Transposons(mean)**	7.26%	2.01%	0.80%
**Transposons(best)**	5.26%	0.20%	0.00%
**Simulated**	8.89%	1.34%	1.29%

The error rate is the average percentage of the bases of a signature, that differ from the consensus of the uncorrected signatures in the ground truth group (i.e. manual assembly copy group) to which the signature belongs. ‘Original’ denotes the uncorrected signatures, ‘corrected’ the corrected signatures. ‘Collapsed variations’ are variations where the majority of signatures of a ground truth group have been corrected towards the incorrect majority base.

**Table 3.** The error rate reduction of the first pass correction

Datasets	Original error	Corrected error	Collapsed variations
**Histone**	8.22%	4.64%	0.19%
**Transposons(median)**	6.51%	4.14%	0.05%
**Transposon(mean)**	7.26%	4.45%	0.12%
**Transposons(best)**	5.26%	2.04%	0.00%
**Simulated**	8.89%	2.49%	1.05%

The error rate is the average percentage of the bases of a signature, that differ from the consensus of the uncorrected signatures in the ground truth group (i.e. manual assembly copy group) to which the signature belongs. ‘Original’ denotes the uncorrected signatures, ‘corrected’ the corrected signatures. ‘Collapsed variations’ are variations where the majority of signatures of a ground truth group have been corrected towards the incorrect majority base.

### Consensus sequence

The final assembly of the histone complex contains 107 copies of the histone repeat sequence and extends over 570 kbp. Two of these copies are shortened by the same long deletion, and two are extended by a doubling of the H2A gene. Finally, another two copies have insertions of different length. The known 4.8 kbp repeat version (8) occurs nine times loosely clustered at the beginning of the complex.

The preprocessing analysis showed 11 reads with a divergent arrangement of two large-scale variations demonstrating the presence of a second haplotype represented by 10–15% of the data, despite the highly inbred strain. These reads have been excluded from all our analyses. Beyond the 3′ end of the complex, we found an assortment of dysfunctional histone copies. The mechanism of this local accumulation of degenerate copies is unknown. No further copies outside the complex were found.

## DISCUSSION

### Correction

There is an intrinsic limit as to what de-noising algorithms can achieve. The presented heuristic is no exception. If the information necessary to correct a certain feature for a certain copy is not part of the data, the variation will be ‘collapsed’, that is, transformed into the majority instance of that particular feature. This collapse is unavoidable and more ‘sharply’ distinguishes between signatures from different copies, in the sense that now all (or most) signatures from this particular repeat copy have the same base at the given position. This means that once the best possible distinction on the basis of the corrected signatures has been achieved, it might be useful or necessary to return to the original signatures for further refinement. A collapse also occurs if error-induced false variations are above the significant threshold. In this case the collapse is just a correction. Table 2 shows that the collapse of variations does not represent a significant problem in our datasets.

The rarer bases at any given SNV position is subjected to a stronger bias by the MSA. The incentive to maximize the count of the most frequent base in every column is inseparable from the MSA algorithm itself. The high insertion rate of PacBio data leads to an abundance of alignment options and as a result the rarer base is often ‘shifted’ out of the correct column. Therefore, rare bases initially show a significantly higher error rate.

Rare bases describe but a few copies of the repeat family. This means that they are more easily collapsed. Indeed, in 1 out of 17 transposon datasets the average recall of the rare bases is clearly worse after the first pass correction. This is due to a number of collapsed variations that decrease the average recall, with their recall of 0.00%. In the corrected version, this number grows to 5 out of 17. Table 5 shows that overall, even the rare bases considerably improve recall.

Rare bases also compete with a high number of false positives, which from an assembly point of view is more problematic than the collapse of variations. Our correction heuristic achieves improved precision for every transposon dataset. Table 6 depicts the overall results for rare base precision improvements.

The collapse of variations may also lead to scenarios in which the average accuracy of corrected bases presents a misleading picture. If a minority base has been fully corrected for one group and collapsed for another group, the average accuracy would be the same, as when both groups show an accuracy of 50%. In the former case however, the internal consistency of both groups is higher, although the overall accuracy is the same. Table 4 shows that while the correction improves the overall accuracy of rare bases, it improves the internal consistency to an even larger degree.

**Table 4.** The internal consistency of groups over all datasets before and after correction

Datasets	Original	First pass	Corrected
**Histone**	91.7%	95.3%	98.8%
**Histone rare**	77.7%	83.4%	91.3%
**Transposons(median)**	93.4%	95.8%	99.2%
**Transposons(mean)**	92.7%	95.6%	98.8%
**Transposons(best)**	94.7%	98.1%	99.8%
**Trans. rare(median)**	85.4%	90.2%	96.8%
**Trans. rare(mean)**	85.7%	89.6%	95.4%
**Trans. rare(best)**	92.7%	95.5%	99.7%
**Simulated**	91.1%	98.5%	99.9%
**Simulated rare**	85.4%	95.8%	99.7%

‘Original’ denotes the uncorrected signatures, ‘corrected’ the corrected signatures, while ‘first pass’ describes the signatures after a single application of the neural network corrector. In the ‘corrected’ and ‘first pass’ cases the internal consistency is the similarity of corrected signatures of a ground truth group (i.e. manual assembly copy group) to the consensus of the corrected group, instead of to the consensus of the uncorrected group. In the uncorrected case, internal consistency is just accuracy, that means 100% - error rate.

**Table 5.** Recall of rare bases

Datasets	Original recall	First pass recall	Corrected recall
**Histone**	77.7%	83.1%	88.6%
**Transposons(median)**	85.4%	87.6%	90.7%
**Transposons(mean)**	85.7%	87.8%	86.0%
**Transposons(best)**	92.7%	95.5%	99.7%
**Simulated**	85.4%	88.1%	90.7%

The percentage of true positives among the correct positives. ‘Original’ denotes the uncorrected signatures, ‘corrected’ the corrected signatures, while ‘first pass’ describes the signatures after a single application of the neural network corrector.

**Table 6.** Precision of rare bases

Datasets	Original precision	First pass precision	Corrected precision
**Histone**	69.0%	79.1%	93.1%
**Transposons(median)**	86.3%	87.0%	91.4%
**Transposons(mean)**	80.0%	81.7%	89.8%
**Simulated**	94.1%	99.0%	99.7%

The percentage of true positives among the positives. ‘Original’ denotes the uncorrected signatures, ‘corrected’ the corrected signatures, while ‘first pass’ describes the signatures after a single application of the neural network corrector.

**Table 7.** Properties of the datasets

Datasets	Copies	Coverage	Length	Variations
**Histone**	107	30–90X	5 kbp	185
**Transposons**	5–157	35–54X	4.3–7.5 kbp	300
**Simulated**	100	50X	5 kbp	300

The number of variations used for correction, is comparable in all datasets, as the histone correction uses additional neighbouring signatures.

### Assembly

We want to emphasize that assembly via clustering including the very specific analyses it entails does not represent a general repeat cluster assembly algorithm. Instead, it illustrates the type of analysis that is feasible via our correction heuristic.

We expect other repeat complexes to exhibit their own idiosyncrasies. It is thus unlikely that our assembly approach will be directly applicable. For instance, all histone reads were oriented according to the template upon extraction by mapping. This simplifies downstream processing, but is only possible because there is no strand reversal in the complex. However, strand reversal does occur, for example, in the Drosophila ribosomal RNA (rRNA)-complex. The rRNA-complex also contains several distinct repeat sequences, which would require further non-trivial adaptations of our clustering algorithm.

While the clustering and graph traversal resolves large stretches of the complex correctly, an important question is how to detect possible misassemblies. Figure 2D shows the assembly graph with misassemblies consisting of collapsed copies indicated by the red nodes. We detect these misassemblies by mapping the clusters that could not be reliably placed onto the assembly which results in coverage anomalies (significantly more than the average 125 signatures per graph layer) in collapsed assembly groups. In Figure 2D the coverage is indicated by both the labels and the diameters of the nodes.

The first misassembly cuts out a large-scale variation by collapsing similar copies at either side of the variation. Starting from this large-scale variation we obtain an assembly which correctly orders the first 90 copies. For the second misassembly this trick does not work, because the collapse already occurs during the clustering phase. Here, we have to resolve the ambiguous part on the basis of a few very long reads.

### Conclusion

To our knowledge this is the first approach that is capable of dealing with the intrinsic complexity of tandem repeat resolution. We expect a wide applicability of the presented methods for the resolution of tandem repeats in genome assembly, but also, for the resolution of non-tandem repeat clusters and long transposable elements.

### Future work

The main current challenge regarding the assembly of further repeat complexes is the initial analysis of the repetitive and unique sequences that occur in the complex as well as the classification of large-scale deletions, insertions and duplications. Automating this task to a significant degree by combining ideas from machine learning with more typical alignment algorithms is a promising direction of future work.

## DATA AVAILABILITY

The code, datasets and assembled sequence are available via GitHub at (https://github.com/PhilippBongartz/DrosophilaHistoneComplex).
